# Regulatory Systems in Bone Marrow for Hematopoietic Stem/Progenitor Cells Mobilization and Homing

**DOI:** 10.1155/2013/312656

**Published:** 2013-06-17

**Authors:** P. Alvarez, E. Carrillo, C. Vélez, F. Hita-Contreras, A. Martínez-Amat, F. Rodríguez-Serrano, H. Boulaiz, R. Ortiz, C. Melguizo, J. Prados, A. Aránega

**Affiliations:** ^1^Institute of Biopathology and Regenerative Medicine (IBIMER), University of Granada, 18100 Granada, Spain; ^2^Department of Human Anatomy and Embryology, School of Medicine, University of Granada, 18071 Granada, Spain; ^3^Department of Health Science, University of Jaén, 23071 Jaén, Spain

## Abstract

Regulation of hematopoietic stem cell release, migration, and homing from the bone marrow (BM) and of the mobilization pathway involves a complex interaction among adhesion molecules, cytokines, proteolytic enzymes, stromal cells, and hematopoietic cells. The identification of new mechanisms that regulate the trafficking of hematopoietic stem/progenitor cells (HSPCs) cells has important implications, not only for hematopoietic transplantation but also for cell therapies in regenerative medicine for patients with acute myocardial infarction, spinal cord injury, and stroke, among others. This paper reviews the regulation mechanisms underlying the homing and mobilization of BM hematopoietic stem/progenitor cells, investigating the following issues: (a) the role of different factors, such as stromal cell derived factor-1 (SDF-1), granulocyte colony-stimulating factor (G-CSF), and vascular cell adhesion molecule-1 (VCAM-1), among other ligands; (b) the stem cell count in peripheral blood and BM and influential factors; (c) the therapeutic utilization of this phenomenon in lesions in different tissues, examining the agents involved in HSPCs mobilization, such as the different forms of G-CSF, plerixafor, and natalizumab; and (d) the effects of this mobilization on BM-derived stem/progenitor cells in clinical trials of patients with different diseases.

## 1. Introduction 

For many decades, bone marrow (BM) transplantation was the only viable method for transplanting hematopoietic stem cells, although their presence had been demonstrated in peripheral blood. Peripheral blood was not used for two main reasons: the number of circulating stem cells that could be gathered by available methods was thought to be inadequate for their autologous and allogeneic transplantation; and the number of contaminated T cells was considered too high for safe allogeneic transplantation [1].

Under steady-state conditions, a small amount of hematopoietic stem cells constantly leave the BM and penetrate tissues, returning to the BM or peripheral niches *via* the blood or lymphatic system [2]. A niche is a subgroup of tissue cells and extracellular substrates that can indefinitely harbor one or more stem cells and control their self-renewal and progeny *in vivo* [3]. The BM niche is strategically placed and organized to support the continuous and balanced production of hematopoietic cells through the strict control of cell survival, self-renewal, and differentiation [4].

The successful transplantation of hematopoietic stem/progenitor cells (HSPCs) is based on their ability to home to the BM niche and on their engraftment capacity. Interactions between HSPCs and their niches are altered during mobilization and must be reestablished during BM homing and repopulation. The homing of HSPCs to BM is a rapid process that takes place during the hours after transplantation and is an essential and necessary requirement for repopulation and engraftment [5].

The use of mobilized peripheral blood is now the method of choice in autologous transplantation for various reasons, including an elevated production of immature cells, and, in comparison to the utilization of BM, the shorter time period required for a satisfactory repopulation, the more rapid engraftment, fewer technical difficulties, lower risk, and considerably less pain [6]. 

HSPCs were used later in allogeneic transplantation [7]. Although BM and peripheral blood are both still considered a source of stem/progenitor cells for this purpose [8, 9], peripheral blood is used in 71% of allogeneic transplantations [6].

Therefore, the regulation of HSPC release from BM and their migration and homing and the mechanism of mobilization pathways involve a complex interaction among adhesion molecules, cytokines, proteolytic enzymes, stromal cells, and HSPCs [10]. The identification of new mechanisms that regulate stem cell trafficking may have important implications for hematopoietic transplants and for cell therapies in regenerative medicine (e.g., for infarcted heart, injured spinal cord, and stroke) [11].

## 2. Regulation Mechanisms for the Mobilization and Homing of HSPCs in Bone Marrow

### 2.1. Factors That Affect Stem Cell Mobilization

*Granulocyte colony stimulating factor* (G-CSF) is the most widely used agent for stem cell mobilization due to its power and lack of severe toxicity. It has two stem cell mobilization mechanisms: firstly, interruption of the anchoring mechanism through downregulation of the expression of stromal cell derived factor-1 (SDF-1) and activation of the CD26 protease that cleaves the SDF-1 N-terminal, impeding binding to CXCR4 by decreasing the function of integrin-*β*1; and secondly, an increase in serum levels of additional cytokines and growth factors [12–14].

Studies of G-CSF in animals with tissue ischemia have demonstrated therapeutic benefits, although with the drawback of a possibly favoring of atherosclerosis [15, 16]. After myocardial infarction (MI), G-CSF promotes the mobilization of cardiac tissue HSPCs and improves the regeneration of cardiomyocytes and blood vessels by the mobilization and subsequent transdifferentiation of BM stem cells. It has been verified that G-CSF avoids H_2_O_2_-induced apoptosis of cardiomyocytes and facilitates cardiac remodeling after MI [17].

However, different studies have demonstrated that the utilization of G-CSF has various disadvantages, including a low therapeutic response and the need for multiple daily injections over several days. These drawbacks can be overcome by combining G-CSF with other cytokines and using different growth factor mobilization strategies [18–20].

*Chemokine CXCL12,* also known as SDF-1*α*, was identified in the supernatant of BM stromal cells; it is expressed at high levels in BM and produced by osteoblasts, endothelial cells, and reticular cells dispersed throughout the BM stroma. It is a potent chemoattractant for HSPCs and has been demonstrated to regulate cell adhesion and survival and cell cycle status [21]. Méndez-Ferrer et al. [22] studied CXCL12 levels in BM, observing that their production follows a circadian rhythm, regulated by the sympathetic nervous system, with noradrenaline acting *via β*2-adrenoreceptors on osteoblasts and *via β*3 adrenoreceptors on nestin-positive stem cells to reduce their production of CXCL12.

*Receptors.* Two chemokine receptors for CXCL12 have been identified (CXCR4 and CXCR7). The presence of CXCR4 on the cell surface bound to other factors promotes migration and homing into or from the BM niche [23, 24]. CXCR4 couples to a series of signaling molecules, stimulating leukocyte chemotaxis and stem cells that express the receptor [11, 25]. The interaction of CXCL12 with CXCR4 in HSPCs is considered an essential signal for regulating HSPC trafficking in BM. Cells without surface expression of CXCR4 are not sensitive to mobilization using CXCR4 receptor agonists or antagonists. One of them, AMD3100, a bicyclam CXCR4 antagonist that is strongly synergic with G-CSF in humans, increases mobilization by one to two logs over G-CSF alone [26, 27]. It is expressed in most types of cancer, including breast cancer, prostate cancer, and kidney clear cell carcinoma [28].

CXCR7 has been identified as a second high-affinity receptor for CXCL12 but does not couple to signaling pathways for migration. It regulates the transendothelial migration of CXCR4+CXCR7+ tumor cells towards a CXCL12 source, an effect that can be blocked by CXCR7-specific antagonists [29]. Upon binding to CXCR7, chemokine CXCL12 is internalized and subsequently degraded; therefore, CXCR7 appears to act as a CXCL12 sink [12]. The two receptors (CXCR4 and CXCR7) interact and can even form functional heterodimers. CXCR4 inhibition does not appear to affect CXCR7 function. Thus, specific blockage of CXCR4 with AMD3100/plerixafor may increase the functions of CXCR7 mediated by SDF-1 [29, 30]. CXCR7 is expressed in cancers of breast, brain, liver, pancreas, lung, and prostate, melanomas, and rhabdomyosarcomas [31, 32].

### 2.2. Mobilization and Homing of Hematopoietic Stem Cells in Bone Marrow and Different Ligands

#### 2.2.1. SDF-1/CXCR4 Axis in Mobilization and Homing

SDF-1 is essential for the circulation, homing, and retention of HSPCs in BM. In 2005, Lapidot et al. demonstrated that SDF-1 is expressed by immature human osteoblasts in the endosteal region [5]. The interaction between SDF-1 and its CXCR4 receptor has been described as a major axis for regulating the migration and mobilization of HSPCs under steady-state conditions [33, 34].

Mobilized human progenitor cells express CD34^+^ and reduced CXCR4 levels, which correlate with greater mobilization, suggesting the participation of SDF-1/CXCR4 interactions in this process. Overexpression of SDF-1 was found to induce the mobilization of stem cells in murine blood [35].

New evidence shows that, in addition to SDF-1, the migration of HSPCs is directed by gradients of the bioactive lipids sphingosine-1 phosphate (SP1) and ceramide-1 phosphate (CP1), which are products of membrane lipid metabolism and involved in stem cell trafficking. This mechanism is based on the significant increase produced by molecules in the chemotactic responsiveness of HSPCs to very low SDF-1 gradients. At molecular level, this sensitization of the responsiveness to SDF-1 depends on the incorporation of the CXCR4 receptor into membrane lipid rafts, activating the complement cascade (CC) [36, 37].

The importance of the CC in HSPC homing has been demonstrated in complement component-deficient mice. Specifically, mice that are deficient in C3 and C5 engraft less successfully with HSPCs from wild-type animals, while HSPCs from C3a receptor-deficient mice show defective engraftment in wild-type littermates. Hence, activation of the CC in BM induces a highly proteolytic microenvironment that degrades SDF-1 [38].

**Figure 1:**
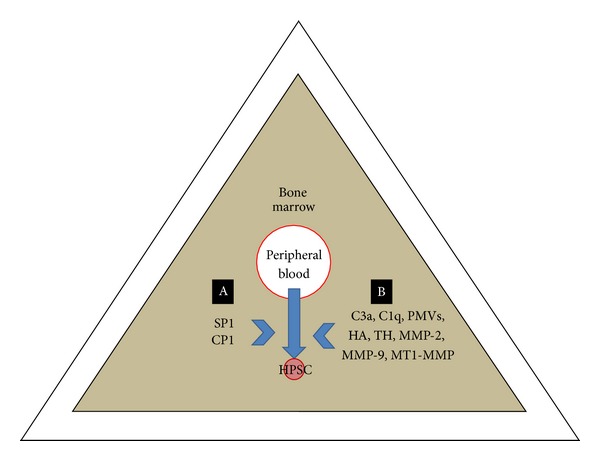
Factors favoring homing of HPSCs in the bone marrow. (A) Chemotactic factors independent of the SDF-1/CXCR4 axis gradient. (B) Triggering or modulating factors of the SDF-1/CXCR4 axis. HSPC: hematopoietic stem/progenitor cells; C3a: complement fraction C3a; C1q: complement fraction C1q; PMVs: platelet-derived microvesicles; HA: hyaluronic acid; TH: thrombin; MMP-2: metalloproteinase-2; MMP-9: metalloproteinase-9; MT1-MMP: membrane type-1 matrix metalloproteinase; SP1: sphingosine-1-phosphate; CP1: ceramide-1-phosphate.

It has been reported that hyaluronic acid (HA) and thrombin (TH) can increase the response of peripheral HSPCs to an SDF-1 gradient. This action may be related to membrane type-1 matrix metalloproteinase (MT1-MMP), increasing its expression and favoring the passage of the HSPCs towards a low SDF-1 gradient [34, 39]. The priming/triggering effect produced by supernatants of leukapheresis products or their components (fibrinogen, fibronectin, complement C1q, complement C3a, platelet-derived microvesicles [PMV], HA, thrombin) was found to be related to an increased secretion of MMP-2 and MMP-9, which, together with the SDF-1-CXCR4 axis, favor the homing of these cells [40] (Figure 1).

#### 2.2.2. Role of G-CSF in the Mobilization and Homing of HSPCs in BM

G-CSF induces the mobilization of HSPCs through the proteolytic inactivation of both CXCL12 and CXCR4 by the granulocyte proteases neutrophil elastase (NE) and cathepsin (CG), which are released in large amounts in proteolytically active form in the BM stroma during G-CSF-induced mobilization. After G-CSF administration, neutrophils show increases in their expression of FcgRI/CD64, CD11b, and FcgRIII/CD16 and in their release of elastase and lactoferrin, allowing neutrophil progenitors to be activated and degranulated directly in the BM stroma during mobilization before their migration into peripheral blood. After their release into the BM environment, these proteases may inactivate a number of proteins essential for retaining HSPCs within the BM, that is, vascular cell adhesion molecule-1 VCAM-1/CD106, chemokine CXCL12/SDF-1 and its CXCR4 receptor (in humans), and c-Kit receptor, all of which may trigger HSPC mobilization [41, 42]. 

Plasminogen (Plg) is a glycoprotein present in blood plasma and in most extracellular fluids as the inactive precursor of a protease enzyme (plasmin) responsible for the dissolution of clots after thrombosis [43]. Various authors have confirmed that Plg plays an essential role in the mobilization of BM stem cells to the peripheral circulation, particularly in G-CSF-induced mobilization of HPSCs. Plg binds to the BM extracellular matrix (ECM) and, after conversion into plasmin, it degrades various proteins of the ECM, including fibrin, laminin, and plasmin. Plg can also activate other proteases, such as MMP-3, MMP-9, MMP-12, and MMP-13, to degrade other matrix components, including collagen [44].

There have also been reports on the importance of urokinase Plg activator (uPA), part of the plasminogen activating system, in the activity of *α*1*β*4 integrin in BM homing. Intact uPA receptors (uPARs) are required for the adhesion and engraftment of HSPCs to BM [45].

#### 2.2.3. Other Ligands

The adhesion molecule Very Late Antigen (VLA)-4 (*α*4*β*1-integrin) is expressed on murine HSPCs and on human CD34^+^ early hematological progenitor cells [46]. Blocking the interaction between VLA-4 and its ligands expressed on BM stroma, by using specific antibodies or small molecule inhibitors, induces rapid mobilization of HSPCs in humans, primates, and mice [47, 48].

One example is the interaction between VLA-4 and VCAM-1 on BM stromal cells, which is essential for BM homing during development and posttransplantation. HSPCs were found to be mobilized by inhibition of this interaction through the administration of function-blocking anti-VLA-4 or anti-VCAM-1 mAbs or through the conditional deletion of either *α*4 integrin or VCAM-1 gene [41].

With regard to the mechanism, VCAM-1 is a substrate of both neutrophil serine-proteases, NE and CG, which accumulate in the BM extracellular fluid during mobilization and cleave VCAM-1 expressed in mouse BM stroma. As a result, the concentrations of soluble VCAM-1 fragments and NE increase in the blood of patients after mobilization. Serine proteases are the sole regulators of VCAM-1 levels in the BM [49]. 

Tyrosine-kinase c-KIT/CD117 receptor plays an important role in mobilization. G-CSF induces the release in the BM of proteases that remove c-KIT receptor (CD117) from the surface of HSPCs. Administration of soluble KIT, which binds to and blocks the endogenous cell factor KIT ligand, in synergy with G-CSF, increases HSPC mobilization. The serineproteases in G-CSF remove c-KIT in small fragments, and its administration to mice reduces c-KIT expression in primitive hematopoietic cells in the BM and peripheral blood. Proteases able to remove c-KIT include NE, CG, proteinase-3, and MMP-9 [50]. Neutrophils are the main source of these proteases, which provide macrophages with a new pathway for regulating the surface expression of c-KIT on HSPCs and may be in part responsible for the downregulation of c-KIT expression on HSPCs mobilized *in vivo*. We highlight that removal of the extracellular domain of c-KIT does not activate its kinase domain; therefore, c-KIT removal during mobilization represents a function loss [51, 52].

## 3. Quantifying HSPCs Mobilization and Homing

Stem cell quantification is usually based on peripheral blood samples, but this method can be challenging. Besides the technical difficulty of precisely determining stem cell mobilization, an increase in stem cells can take place in non-BM tissues, such as splenic or adipose tissue [41].

Various factors can influence the quantification of mobilized stem cells. One example is the daily variations in BM SDF-1*α* levels, which means that the stem cell count may vary according to the time of day that the sample is drawn [22]. In addition, HSPCs mobilized with CXCL2 are less dependent on CXCR4 in comparison to those mobilized with G-CSF, which are in turn more dependent on selectins and integrins [53]. After mobilization, stem cells may rapidly home back to the BM, but they may also redistribute to other tissues with local stem cell niches, such as the liver, spleen, lungs, myocardium, and adipose tissue [54–57]. Thus, the count of mobilized stem cells in peripheral blood may include not only those from the BM but also those from these other organs*.*

Recent studies demonstrated that the mobilization of different subpopulations of progenitor cells in the BM can be directly and accurately counted, allowing the effectiveness of different mobilizing agents to be compared over the short and long term [58]. In these experiments, instead of drawing peripheral blood samples, an *in situ* perfusion system is placed in the hind limb of the mouse, allowing specific counts to be made of the total number of hematopoietic progenitor, endothelial and mesenchymal cells mobilized by the BM during a given time period.

It has been reported that the number of HSPCs is inadequate in around 20% of patients treated with G-CSF for BM transplantation. Successful hematopoietic reconstitution requires the transplant of at least 2 × 10^6^ CD34^+^ cells/kg, and a higher number is associated with a lower incidence of graft-*versus*-host disease [59, 60].

## 4. Therapeutic Outcomes: HSPC Mobilization

In the clinical setting, HSPC mobilization from the BM to blood has been used for stem cell transplantation and to stimulate angiogenesis in ischemic tissues [61]. Factors that limit the therapeutic potential of HSPCs include advanced age and cardiovascular risk factors, including hypercholesterolemia, hypertension, and smoking [62].

### 4.1. Current Standard Agents

Since the early 1990s, G-CSF has been the most widely used agent to mobilize HSPCs for BM transplants [63]. The administration of exogenous G-CSF increases the production of neutrophils by the BM, inducing a rapid exit of HSPCs. Numerous studies have compared the effectiveness of different forms of G-CSF (pegfilgrastim, pegylated form; filgrastim, nonglycosylated form; lenograstim, glycosylated form), which are yet to be well defined, and they found little difference among them [64–66]. Filgrastim (or lenograstim in some countries) remains the agent of choice for the mobilization of allogeneic peripheral blood stem cells from normal donors [67]. The Food and Drug Administration approved pegfilgrastim for reducing the duration and severity of the neutropenia associated with many chemotherapy regimens.

A highly significant positive correlation has been found between the concentration of CD34^+^ cells before apheresis in peripheral blood and the predicted quality of collections from one-to-three leukaphereses [68–72]. The most frequent G-CSF dose for mobilization in healthy donors is 10 *μ*g/kg/day subcutaneously from day 5 until sufficient CD34^+^ cells are collected. Various authors found no increase in stem cell yields at higher G-CSF doses [67, 73]. Although CD34^+^ cell mobilization is increased by its administration twice daily [74], a single dose is preferred [75]. G-CSF is generally well tolerated, and the most commonly observed adverse effects are bone pain, fatigue, nausea, and headache [76, 77]. In a large retrospective study of mobilization in 85 healthy donors, a yield of >85% CD34^+^ cells was more frequently obtained in the afternoon than in the morning [78]. It should be borne in mind that G-CSF-mobilized HSPCs are different from those present in the BM under normal conditions and express lower levels of c-kit, VLA-4 integrin, and CXCR4 [61].

Most centers use G-CSF alone or in combination with chemotherapy in mobilization regimens [79, 80]. A higher CD34^+^ cell yield is obtained with G-CSF plus chemotherapy than with G-CSF, besides the antitumor effect [81, 82]. In most patients, the mobilization procedure is started with G-CSF after the 2nd or 3rd cycle of chemotherapy treatment. In patients with multiple myeloma (MM) or non-Hodgkin lymphoma (NHL), cyclophosphamide is followed by the administration of 5 *μ*g/kg G-CSF [83]. Higher doses of chemotherapy have been associated with a greater frequency of platelet transfusion and hospitalization for febrile neutropenia [82].

It has been estimated that the incidence of poor mobilizers ranges from 5% to 40% of healthy donors and patients [71, 84, 102, 103]. G-CSF has been reported to fail to mobilize a sufficient number of PBSCs for transplantation in some elderly patients and especially in patients with a history of chemotherapy or radiotherapy [69, 84, 85, 93, 97, 103]. Other factors associated with poor mobilization include a low platelet count immediately before mobilization [85], baseline thrombocytopenia [85, 92], diabetes [104], and a low TNF-*α* level [105]. However, it is difficult to predict mobilization in donors due to the absence of well-established predictive factors [68–71, 84–101], and there is no consensus on the definition of poor mobilizers [69].  Table 1 lists variables that have been associated with mobilization failure risk. Olivieri et al. and the “Gruppo Italiano Trapianto di Midollo Osseo” [87] recently attempted to clarify the definition of “poor mobilizers” in lymphomas and multiple myeloma patients. They proposed a peak of CD34^+^ cells of >20 *μ*L in peripheral blood before collection as a reliable indicator of satisfactory mobilization capacity. Poor mobilization has important consequences, increasing the morbidity after repeated mobilization attempts and significantly reducing the possibility of transplantation [66].

### 4.2. Novel Mobilizing Agents

Plerixafor (AMD3100) has emerged as a promising HSPC mobilization agent. It is a reversible CXCR4 antagonist and produces the fast release of stem cells from BM niches into the blood stream [54, 106]. High expectations have been raised by this agent in the setting of PBSC transplantation [69, 106], because of its ability to mobilize large numbers of CD34^+^ cells in patients with a poor response to G-CSF administration [54, 107, 108]. The combination of plerixafor with G-CSF produces even greater increases in circulating CD34^+^ cells [109]. It is also effective in patients that have previously received chemotherapy, and it acts in synergy with G-CSF and chemotherapy [54, 66, 107]. In December 2008, the United States Food and Drug Administration approved plerixafor in combination with G-CSF for HPSC mobilization in patients with NHL and blood stream undergoing autologous peripheral blood hematopoietic cell transplantation [67].

The recommended standard dose of plerixafor is 0.24 mg/kg/day by subcutaneous injection, with adjustments in particular cases such as myeloma patients with advanced renal failure [110]. Plerixafor used alone rapidly mobilizes HSPCs, reaching a maximum at 6–9 h, and it improves the yield in healthy donors either as a single agent or in combination with G-CSF [30]. Recent research by Abhyankar et al. [111] confirmed that superior results are obtained when plerixafor is given at 5:00 PM the evening before apheresis. The most common adverse effects of plerixafor are mild-moderate gastrointestinal reactions, injection-site reactions, and paresthesias [112].

G-CSF plus plerixafor was more effective for first-line mobilization than G-CSF alone in MM and NHL patients [113] and also proved effective in patients with Hodgkin's lymphoma (HL) [114, 115]. Various studies found that this combination can safely and effectively remobilize NHL patients in whom previous mobilization approaches have failed [116, 117]. This combination may be of special value in heavily pretreated patients [118]. 

There is still little experience of mobilization regimens using plerixafor in combination with chemotherapy plus G-CSF [119, 120]. Plerixafor may be useful for HPSC mobilization in patients needing high chemotherapy doses as well as in patients with risk factors for poor mobilization, for example, age, history of radiotherapy or exposure to fludarabine, or lenalidomide, among others [72]. Yannaki et al. [121] proposed G-CSF or plerixafor as mobilizers in nonsplenectomized adult patients with thalassemia and plerixafor in splenectomized thalassemic adults. It has also been suggested that HPSC mobilization would be improved by plerixafor combined with G-CSF and pegylated-G-CSF after chemotherapy in patients with advanced germ cell cancer [122] as well as in those with MM or lymphoma. 

For the appropriate use of immediate salvage plerixafor, it is critical to measure real-time indicators of poor and slow mobilizers during mobilization treatments. It is indicated when the concentration of CD34^+^ cells is <5-6/*μ*L on day 4 of G-CSF apheresis [123]. Awan et al. [124] administered salvage plerixafor in patients failing chemotherapy and G-CSF mobilization and obtained ≥2 × 10^6^ CD34^+^ cells/kg in all cases. Although plerixafor represents an advance in HSPC mobilization, 30% of patients that fail with G-CSF protocols also fail with G-CSF plus plerixafor, which appears to be attributable to a low or defective reserve of HSPCs or niche problems. Greater understanding of the molecular mechanisms underlying the action of these factors will allow the design of predictive algorithms and adequate mobilization protocols in the future [66].

The *α*4 integrin antibody (CD49d) natalizumab, another proposed agent, achieves adequate cell mobilization in patients with a poor response to G-CSF and plerixafor [66, 125, 126]. Natalizumab is a recombinant humanized IgG4 monoclonal antibody that binds to the *α*-4 subunit of the *α* 4-*β*1 integrin and inhibits the *α*-4-mediated adhesions of leukocytes to their counterreceptors. It has been used in the treatment of multiple sclerosis (MS) and Crohn's disease. In relapsed MS patients, a single dose (300 mg) of natalizumab produced a 5-fold increase in circulating CD34^+^ cells one day later [126]. POL 6326, a CXCR4 antagonist, was studied in MM patients and healthy volunteers and proved to be well tolerated and effective in the mobilization of CD34^+^ cells [127]. Recent results showed that the addition of BKT140 (4F-benzoyl-TN14003), another CXCR4 antagonist, to G-CSF can increase the mobilization of CD34^+^ cells and reduce the number of aphereses. BKT140 has also shown an antitumor effect, increasing apoptosis in human-derived MM, lymphoma, and primary leukemia cells, although further research is required to establish its anti-MM effects [128].

### 4.3. Stem Cell Therapy in Ischemic Heart Disease

Over the past few years, interest has grown in the application of stem cell therapy in ischemic heart disease. In myocardial repair, stem cell homing signals play a decisive role in mobilizing BM stem cells towards the ischemic area of the heart. The therapy is designed to enhance the homing, survival, persistence, and differentiation of stem cells in the infarcted area, and the chemokine SDF-1*α*/CXCL12 has proven to be the most potent stem cell homing factor [129]. Research by Wang and Luther [130] on the infarcted heart showed that hypoxic preconditioning activates SDF-1*α*/CXCR4 signaling and upregulates vascular/angiogenic factors that mobilize progenitor cells. SDF-1*α* secretion in the infarcted heart creates an environment that enhances the homing of circulating CXCR4+ stem cells and other stem cells. BM-derived mesenchymal stem cells have shown good results in post-MI cardiac repair [131]. In a study of patients with acute MI, Karapetyan et al. [132] found that the bioactive sphingophospholipids, SP1 and CP1, regulate trafficking of HSPCs. Stem cell-based and microRNA (miRNA, miR) based therapeutic strategies appear to offer a promising perspective for patients with cardiovascular disease, especially MI [133].

## 5. Stem Cell Mobilization in BM and Clinical Trials 

Clinical trial NCT00536887 (Effects of atorvastatin 10 mg versus 40 mg in eight-month followup coronary flow reserve and bone marrow stem cell mobilization in patients with acute myocardial infarction) demonstrated that different doses (10–40 mg) of atorvastatin were effective to enhance BM stem cell mobilization in patients with acute MI, increasing the mobilization of CD34^+^ and CXCR4+ cells, reducing cytokine levels and regenerating microvascular integrity [134]. Although this trial ended in 2008, the final results had not been published at the time of writing this review.

Another clinical trial in acute MI patients (NCT00126100: Bone marrow stem cell mobilization therapy for acute myocardial infarction [REVIVAL-2]), which was completed, reported that transplantation of blood-derived or BM-derived progenitor cells can improve cardiac regeneration and that G-CSF induces BM stem cell mobilization and increases the number of circulating stem cells available for this purpose [135, 136].

The Gregorio Marañón Hospital in Spain is running a Phase 2 clinical trial (NCT00984178: Trial of hematopoietic stem cells in acute myocardial infarction [TECAM2]) to compare the effectiveness of four strategies to prevent postinfarction ventricular remodeling: conventional treatment for reperfused extensive acute myocardial infarction; intracoronary transplantation of autologous bone marrow stem cells; mobilization of bone marrow stem-cells induced by G-CSF; and the combination of stem-cell transplantation with G-CSF-induced mobilization. This trial is currently recruiting participants, and no date has yet been given for the end of the study.

Clinical trial NCT00001071 (A study of stem cells and filgrastims) was carried out in patients at various stages of HIV-1 infection and in HIV-negative volunteers and investigated the safety of stem cell harvesting after using filgrastim (G-CSF) to mobilize BM stem cells into the peripheral blood. This study, which has ended, found that the mobilization and harvesting of bone marrow progenitor cells from persons infected with HIV-1 induced a transient increase in viral replication in some patients but was not associated with adverse effects [137, 138]. 

Clinical trial NCT00011830 (Stem cell mobilization potential in patients with aplastic anemia in remission) studied the use of filgrastim in patients with aplastic anemia (aged ≥12 years) in remission after successful treatment with immunosuppressive drugs.

It investigated whether G-CSF administration generates sufficient BM-produced cells that mature into white/red blood cells and platelets for use in future treatments and whether successfully treated patients who then relapse can benefit from autologous stem cell transfusion. G-CSF was s.c. injected daily for up to 10 days. Stem cells were collected by apheresis, usually after 5 or 6 days of filgrastim injections. The results of this trial have not yet been published. 

## 6. Conclusions 

HSPCs are mobilized from the BM in various situations, including hematopoietic transplantation, AMI, bone marrow injuries, and stroke, among others. Researchers have demonstrated that regulation of the mobilization and homing of HCPCs from the BM plays a critical role in repairing damage to different tissues. Various factors influence the regulation mechanisms for HSPC mobilization and homing from the BM, including SDF-1 and its CXCR4, which have been implicated as a major pathway for regulating the migration and mobilization of HSPCs under steady-state conditions. G-CSF induces HSPC mobilization through the proteolytic inactivation of CXCL12 and CXCR4 by NE and CG and through the interaction of other ligands such as VLA-4 and VCAM-1 in BM stromal cells. It is now possible to accurately quantify the mobilization of stem cells by direct measurement in the BM, allowing comparison of the efficacy of different agents over the short and long term. Mobilizing agents being used in different diseases include the distinct forms of G-CSF and, especially, plerixafor, which has represented a major advance in novel strategies for HSPCs mobilization, especially in patients with a history of mobilization failure. Various clinical trials are under way to evaluate the effectiveness of different factors for the mobilization of BM stem cells.

## Figures and Tables

**Table 1:** Variables associated with increased risk of possible mobilization failure.

Variable	Main references
Age	Hosing et al., 2009 [84] Kuittinen et al., 2004 [85] Wuchter et al., 2010 [86] Olivieri et al., 2012 [87]

Mobilization with G-CSF alone	Hosing et al., 2009 [84] Petit et al., 2002 [88] Bensinger et al., 2009 [89]

Bone marrow infiltration by tumor cells	Kuittinen et al., 2004 [85] Demirer et al., 1996 [90]

Disease	
Lymphomas > myeloma	Pusic et al., 2008 [91]
Chronic lymphocytic leukemia	Jantunen et al., 2008 [92]
Disease status	Haas et al., 1994 [93] Wuchter et al., 2010 [86] Bensinger et al., 2009 [89]

Previous myelotoxic chemotherapy	Mohty and Ho, 2011 [69] Gertz et al., 2010 [71] Jantunen et al., 2008 [92] Wuchter et al., 2010 [86] Bensinger et al., 2009 [89] Lysak et al., 2005 [94] Laszlo et al., 2004 [95] Popat et al., 2009 [96]

Previous extensive radiotherapy to BM	Bensinger et al., 1995 [70] Haas et al., 1994 [93] Sevilla et al., 2013 [97] Demirer et al., 1996 [90] Olivieri et al., 2012 [87]

Low premobilization BM cellularity	Hosing et al., 2009 [84] Olivieri et al., 2012 [87]

Low baseline CD34^+^ cell count	Fruehauf et al., 1999 [68] Han et al., 2012 [98] Fu et al., 2006 [99]

Low platelet count before mobilization	Fruehauf et al., 1999 [68] Hosing et al., 2009 [84] Wuchter et al., 2010 [86] Han et al., 2012 [98] Suzuya et al., 2005 [100] Zubair et al., 2008 [101]

G-CSF: granulocyte colony-stimulating factor.

BM: bone marrow.

Myelotoxic chemotherapy: melphalan, carmustine, dacarbazine, fludarabine, lenalidomide, platinum compounds.
